# Supplementary material for: Pigment Identification and Gene Expression Analysis during Erythrophore Development in Spotted Scat (Scatophagus argus) Larvae
Source: Int J Mol Sci. 2023 Oct 19;24(20):15356. doi: 10.3390/ijms242015356 (PMC10607709; doi:10.3390/ijms242015356)
Supplement: Supplementary file 1 [file ijms-24-15356-s001.zip › ijms-2617754-supplementary.pdf]

Supplementary Materials

Table S1. Primers for qRT-PCR validation.

Gene Name	Primer Sequence (5′- 3′)	Product Size (bp)
soat2	CGGTCATGCTGGTGACCATA<br>ACTGAGCTGATCCTGGACCT	112
rlbp1b	CCCTGGTACTTCACCACCAC<br>GCCATCGTACTTGGGAGCTT	165
rbp1	CTGTCTGGAGTGGATGACAGG<br>GGTCCAGCCTCTTCCTTCAA	102
apoa1	GTCAAGCTGGAGCCCGTAAT<br>AGGGACTTGCCATCTGCTTC	148
rpe65a	GACTGCTTCGAGTCCAACGA<br>CAATGGCTGCCCCTTTGAAC	100
rh2	AGGTACCTTCCTGAGGGCAT<br>CGGGAACGAAGAAGTGGACA	118

Table S2. Primers for candidate genes of red coloration.

Gene Name	Primer Sequence (5′-3′)	Product Size (bp)
plin2	TGGGTTCACTGTCCACCAAG<br>CACACCACCAGGGAGCTAAG	112
scarb1	TTGAGCCGTCGATGTCTGTC<br>CAACAACCGCAACACGTAGG	113
bco1	CTGTCTGGAGTGGATGACAGG<br>GGTCCAGCCTCTTCCTTCAA	132
plin6	CGTTACCCTTTGCTGGGTCT<br>AACCATCACCTCGTCTGCTG	204
csf1ra	CTTCAGTGGAATGGAGGCGT<br>CGTCTGTGTTTGGGTAGCCT	151
gch2	GGGCTATCACGAGACCATCG<br>TTGAACCTGAAGCCTCCGAC	226
slc2a15b	GCAGGAATCCCACAACCGTA<br>ACGCTGATGTAACGCAGGAA	212
apoda	CCCTCCGTTCAATCAGCCTT<br>ATTTCGGAGCTGACCACTCG	152

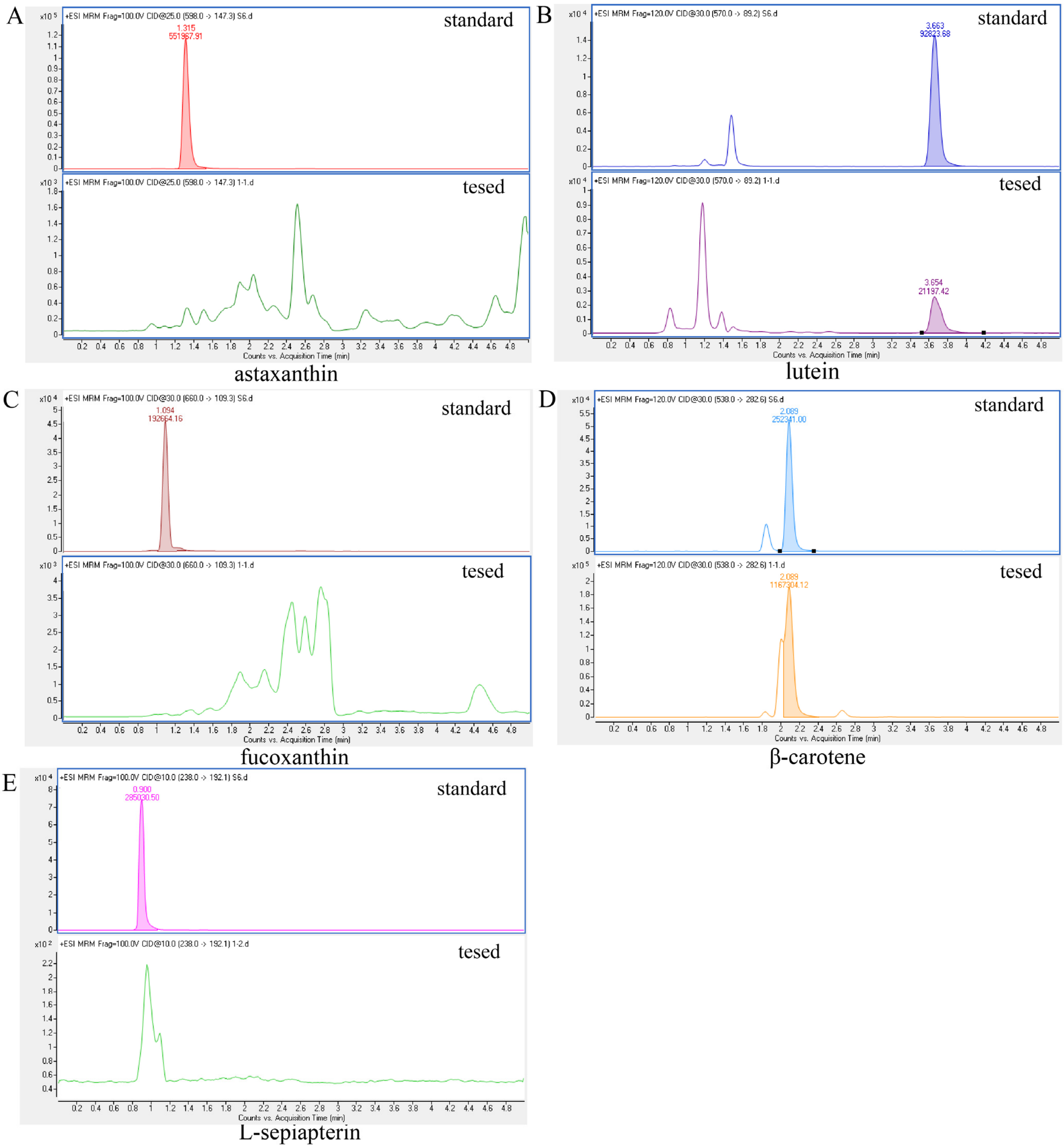

Figure 1. Pigments detected by LC-MS. Identification of pigment types responsible for the red coloration by LC-MS. "Standard" refers to the pigment standards, and "Test" represents the spotted scat larvae sample. **A** represents astaxanthin, **B** represents lutein, **C** represents fucoxanthin, **D** represents β-carotene, **E** represents L-sepiapterin.
